# Replication and Reproduction: Crises in Psychology and Academic Labour

**DOI:** 10.1177/10892680211055660

**Published:** 2022-02-24

**Authors:** Felicity Callard

**Affiliations:** 1 3526 University of Glasgow, Glasgow, UK

**Keywords:** academic labour, history of psychology, invisible labour, university, replication crisis, social reproduction, work

## Abstract

Discussions of the replication crisis in psychology require more substantive analysis of the crisis of academic labour and of social reproduction in the university. Both the replication crisis and the crisis of social reproduction in the university describe a failure in processes of reproducing something. The financial crisis of 2007–8 shortly preceded the emergence of the replication crisis, as well as exacerbated ongoing tendencies in the organisation and practices of university research (particularly the use of precarious contracts and the adjunctification of research). These provide two reasons to address these two named crises together. But many analyses of and responses to the replication crisis turn to research culture, often at the expense of adequate investigations of research labour. Today’s psychological sciences are made through multiple forms of labour: these include researchers, who range from senior principal investigators to sub-contracted, and exploited, research assistants; research participants/subjects, who include those providing labour for experiments via exploitative platforms including Amazon Mechanical Turk; and workers providing heterogeneous technical and administrative labour. Through understanding what is at stake for these multiple forms of labour, psychology might better analyse problems besetting psychology today, as well as develop different imaginaries and practices for how to address them.

## The Labour of Replication

‘Is psychology suffering from a replication crisis? What does “failure to replicate” really mean?’ (Maxwell et al., 2015)
‘What does it mean to say there is a crisis in academic labor?’ (Moten & Harney, 1999)

In the first quotation above, Scott Maxwell and colleagues survey the scene in psychology in the second decade of the 21st century and ask why many experimental results appear to fail to reproduce themselves. In the second quotation, Fred Moten and Stefano Harney survey the scene of academic labour at the very end of the 20th century and ask, ‘Why did this generation [of the 1960–80s] fail to reproduce itself?’ Both questions wonder about what causes the processes of reproducing something to fail: in the first case, a repetition of an experiment in the service of corroborating results; in the second, a process of social reproduction through which conditions of labour and consciousness are remade within the university. What might be gleaned by placing these two questions – which both address reproduction and their apparent failure – alongside one another? Replication and reproduction name what repeats; what recurs. Replication describes the process of reproducing something, and reproduction, like replication, can involve the producing of phenomena in the form of a copy.^1^ My contribution to this special issue on the replication of crises departs from the observation that while replication and reproduction are intimate neighbours, replication in psychology and (social) reproduction in the university have not adequately been considered together, even as both are assumed, by many, to be ‘in crisis’. One good indicator of their general distance from one another is that precarity, casualization, exploitation and working conditions within the academy (Brienza, 2016; Carpenter et al., 2021; Kezar et al., 2019; Loveday, 2018; Peacock, 2016; Thorkelson, 2016) are rarely a central focus of debates and writings addressing the replication crisis in and beyond psychology. I sketch out some of the reasons why this might be the case and why it is worth thinking (experimental) replication and (social) reproduction together. Bringing these literatures more closely together will help us understand not only which concepts and frameworks are – and aren’t – being used to address the ‘replication crisis’ in psychology, but will also provide greater clarity in understanding which modes of socio-political intervention might – and might not – help address phenomena that are considered to be in a state of crisis. It will also clarify how the epistemological domain of psychology imagines, writes about and indeed constitutes itself, today. Another way of saying all this is that if you are worried about reproducibility in psychology, you should be worried about current conditions undergirding the reproduction of labour in the university.

Nick Mitchell, in his writings on the university, has demonstrated how the ‘materiality of the labour practices’ underlying the production of knowledge shapes the form that epistemic domains take (Mitchell, 2015, 2019; undercommoning, 2016). Following Mitchell opens up different ways of understanding disciplinary histories. Mitchell writes in particular about the formation of the discipline of ethnic studies in relation to the site of the university: the emergence of ethnic studies in the United States, Mitchell insists, relied on an early form of adjunct labour. In recounting this history, Mitchell undercovers what is at stake in how this field, and its history, is envisaged: ‘forms of fantasy’ consolidate one part of a history – namely, the radicality of ethnic studies – but occlude the reliance on adjunct professors that underpinned the field’s emergence (undercommoning, 2016). The materiality of labour practices has been excised from accounts of the solidification of ethnic studies in the university; so, too, has the materiality of labour been largely kept out of discussions of the ‘replication crisis’ in psychology. The task is to grasp the work that keeping the ‘labour ecology of today’s academy’ (Mitchell, 2019) out of consideration does. How does it shape what the replication crisis is imagined to be, as well as the potential routes or interventions that are envisaged as able to ameliorate or resolve it? I explore two dominant and related fantasies underpinning discussions of the replication crisis: (i) that the generic and universalized figure of ‘the psychologist’ is the appropriate protagonist and focal point for these discussions; and (ii) that much of the work of the psychological sciences is imagined to take place without workers.

## The Labour of Research

The ‘replication crisis’, as a phenomenon and an object of analytic concern, has been defined and analysed by multiple actors. There is neither wholehearted agreement on the lineaments of this crisis nor, indeed, on whether there is a crisis.^2^ The phrase ‘replication crisis’ is usually used to signal concern that the findings from many experimental studies appear to be unable to be reproduced: this, for some, places psychology’s experimental robustness in doubt. While much of the focus of concern has been on questions of psychological method, analyses of the replication crisis have demonstrated how broad epistemological and ontological questions are also at stake. Deliberations over how psychology comes to know, as well as how psychology envisages its scientific objects, entail deliberations over professional norms and values, since norms and values guide epistemological and ontological assumptions, and vice versa (Flis, 2019; Morawski, 2019; Wiggins & Christopherson, 2019). Writings addressing the replication crisis thereby intersect with a broad set of literature on current arrangements governing and infrastructures surrounding university research (e.g. the proliferation of rankings, metrics and other evaluative techniques (Burrows, 2012), the pressure to acquire grants (Lilienfeld, 2017) and practices of academic gaming (Biagioli et al., 2019)). Diverse actors who wish to intervene to ameliorate or resolve the crisis have therefore proposed a series of changes to the operations and overarching governance of the psychological and other sciences – as well as to their methods. These include practices of open science, the increasing of transparency, improvements in scientific efficiency, corrections in relation to the use of statistics, prescriptions for how replication studies ought to be undertaken, requirements for certain practices of reporting, improvements in scientific training, and shifts in the incentives and plaudits that surround scientific research (e.g. Button et al., 2013; Munafò et al., 2020; Nosek et al., 2015; Tackett et al., 2019). At the heart of many of these efforts to improve scientific practice lies the figure of the psychologist herself.

To provide scaffolding for the argument I pursue in the remainder of this article, I take up the writings of Jill Morawski, historian of psychology, on the replication crisis. Morawski has provided some of most astute assessments of how the subjectivity of the psychologist – and in particular the psychology of the psychologist – figures disproportionately in those debates (Morawski, 2019; 2020; see also Flis, 2019). Not only is the psychologist considered to be subject to cognitive bias (e.g. confirmation bias, hindsight bias), but in fact many of her intimate characteristics (her ‘cognitions, emotions, lived experiences and behaviours’) might well contribute to the problems described under the phrase *replication crisis*. Morawski argues:

Intertwined with technical matters of research methods and normative conduct, assessments of psychologists form part of a conceptual web that links a belief that replication is the “cornerstone” of science with a related belief that objectivity is its foundation. In turn, these epistemic premises are connected with beliefs about psychologists’ psychology and allied conceptions of psychologists as moral and public actors. (2020, p. 178)

Psychologizing the psychologist is one of the motors through which the replication crisis in psychology keeps turning: Morawski notices how many ‘appraisals … target, both directly and indirectly, problems of the researcher as individual actor’ (Morawski, 2020, p. 177). The figure of the psychologist must be exhorted, encouraged and incentivized to act differently – even as many of the psychological attributes that are understood to be problematic are difficult to shift.

What is noticeable for Morawski, in current writings addressing the replication crisis, is the absence of cultural analyses of the crisis (i.e. analyses that would place this scientific crisis in a broader cultural context). For Morawski, such an absence ‘might be itself symptomatic’ (Morawski, 2019, p. 233). If you agree with Morawski that something symptomatic might be at work – and I do – the question then arises: Symptomatic of what? Morawski contends that this lack of cultural analysis is perhaps not unconnected to the ‘dire cultural climate in which [the current crisis] is transpiring’ (Morawski, 2019, p. 221). She links concern about ‘unreliable knowledge and questionable research’ to the financial crisis of 2007–2008 and its sequelae (including the coming to light of doubtful, at times criminal financial practices), as well as increased contestation over what constitutes a fact. Here, Morawski extends a hypothesis that others, elsewhere, have attempted to investigate empirically. John Walsh and colleagues, for example, have explored whether countries with ‘higher rates of societal (business, government) corruption have higher rates of pathologies in science’; they make reference to how high stakes incentives can lead to excessive risk tasking (and here they mention estate agents, bankers and mortgage bankers prior to the financial crisis) (Walsh et al., 2019). I welcome further investigations of a potential relationship between the emergence of the ‘replication crisis’ and the financial practices that accompanied the financial crisis of 2007–2008. But what I am struck by here is what is still not explicitly named, either by Morawski or indeed by other authors writing on the replication crisis. That is the consequence for labour of that crash and its aftermath – as well as the potential bearing of those consequences on the emergence, persistence and very shape of a ‘crisis of replication’.

The financial crisis exerted profound and ongoing aftershocks on the ecology of research inside the university and in para-university research spaces. Decreases in public expenditure on research following the crisis (OECD, 2021) were accompanied, in many countries, by austerity policies and labour reforms that exacerbated on-going tendencies in the organisation and practices of university research. These increased and intensified: the use of precarious contracts and the adjunctification of research; workloads and productivity expectations; the ‘projectification’ of research; the introduction of new forms of digital labour and the further entanglement of private companies in the assemblage making up higher education (Herschberg et al., 2018; Komljenovic, 2019; Schwaller, 2019). It is difficult to track how exactly those working in the psychological sciences have been affected, not least because data are not always available to understand shifts in the use of postdoctoral, fixed-term and other kinds of casualized positions, nor what those inhabiting those positions go on to do (Woolston, 2020). Research from the United States National Postdoc Survey published in 2018 indicates that in the US, only approximately 15% of those in post-doctoral positions (across all disciplines) go on to tenure-track positions (McConnell et al., 2018). In relation to psychology specifically, the US College and University Professional Association for Human Resources reported that the growth of non-tenure track teaching positions in psychology was slower than it was in many other disciplines: while from 2014 to 2019 non-tenure-track full-time teaching faculty increased steadily as a percentage of all faculty (moving from being approximately a quarter to being approximately one third), non-tenure-track faculty in psychology made up 22% of faculty positions in 2019 and had increased proportionally less quickly (Bichsel et al., 2019). (We should note that these figures do not include non-tenure research faculty.) More broadly, as Bennett Carpenter et al. (2021), amongst others, have argued, higher education is experiencing a particular crisis of social reproduction: many university systems are increasingly ‘sites of accumulation’, and certainly cannot be assumed – as they often were, post-war – to be acting predominantly as sites of production for a future workforce or in relation to national culture.^3^ This has meant the creation of revenue streams that require high levels of indebtedness in students, as well as high levels of exploitation, casualization and unemployment amongst many employees (Carpenter et al., 2021; Torracinta, 2020). There are many ways in which such processes – and the shifts in work practices that accompany them – might intersect with the concerns raised by those writing about the replication crisis. For example: How do such processes affect how data are collected, analysed, stored and shared, and broaden the kinds of actors who gather and have access to such data? Or how research participants are recruited, consented into research and compensated? Or shift where time and focus is – and is no longer – able to be spent in the carrying out and reviewing of scientific research? Or affect how spatially and temporally distantiated researchers finds ways, as they collaborate, to ensure the comparability of data, or standardise modes of measuring and recording? Or intensify hierarchical relationships that are embedded through unequal contract arrangements, and which hence potentially change collaborative practices and modes of communication?

The labour required to design, carry out, analyse and disseminate psychological research is, moreover, multiple. The labour of psychological researchers themselves is intensely heterogeneous: researchers range from senior principal investigators, to stably employed academics, to serial postdoctoral researchers, to doctoral and pre-doctoral students, to contracted out and zero-hour-contract research assistants. (And let us leave to one side the numerous sites beyond the university, and para-university research spaces, where psychological research is being undertaken.) The labour of research participants/subjects is also required for the production of psychological research, as well as for disciplinary reproduction. And, crucially, today’s psychological research assemblage requires multiple other kinds of work, both technical and administrative, which is often not envisaged as part of research, though is fundamental to it (Barley & Bechky, 1994). These require many different kinds of skill; are often spatially stretched over significant distances and country borders that cross the Global South and Global North; are often mediated through multiple intersecting infrastructures (including complex networks that bring together information technologies, artificial intelligence, metrics and databases and publishing); and often involve actors, including corporate actors, in and outside the university. Morawski (2020) and Flis (2019) have rightly diagnosed that there is ample reflection on how psychologists’ *psycholog* might affect the conduct of scientific research, and hence might bear on matters that affect reproducibility. But there is little similar attention to the materiality either of the psychologist’s labour (physical, mental, emotional), or of the labour of those with whom she requires contact, directly or indirectly, in the pursuit of scientific research.

The replication crisis, Morawski argues, shows up an intimate relationship between questions of epistemology and questions of ethos: how scientists know is deeply connected to how scientists think about themselves as scientific selves (see also Anderson, 2008; Galison & Daston, 2008). But if we are to understand how the epistemic constraints and norms that govern psychological research are themselves attached to particular kinds of social formations, we need to attend more carefully to how such social formations are shaped through diverse kinds of labour relations. If questions of ethos and epistemology foreground considerations of morals, values and norms, these do not float free from the labour relations through which they emerge and in which they are entangled (see, for example, Bigg, 2000). But discourse surrounding the replication crisis, when addressing questions of epistemology and ethos, disproportionately addresses behaviour and not labour relations. We find concerns with behaviours, which are understood to be nested within, and often responsive to, particular facets of what is termed scientific culture. Behaviours are, moreover, often understood through frameworks both psychological and (neoclassical-)economic. As Morawski has noted, it is understood to be ‘self-interested maximizing’ behaviour that produces scientists’ problematic behaviours, such as competitive struggles over publishing eye-catching findings, or the ‘salami-slicing’ of research findings to maximize the number of publications (Morawski, 2020, p. 186).

Such neo-classical economic and psychological frameworks narrow the horizons of what is envisaged as malleable and re-makeable. Incentive structures can be altered, and indeed should be altered, to push particular behaviours in a different direction. Vincent Larivière and colleagues, in their analysis of authorship, emphasise the importance of contributorship and contributor statements, given the growing fragmentation of tasks that characterizes collaborative research (Larivière et al., 2016). Serge Horbach and colleagues, in an empirical investigation of the reporting of research misconduct note the role of power relations in the reporting of such misconduct, pointing to how ‘younger researchers, researchers with temporary appointments and those in lower academic positions are less likely to act and report than their senior and permanently appointed colleagues’ (Horbach et al., 2020). But while such research does consider discuss divisions of labour, it shies away from investigation of the material underpinnings and consequences of such division of labour. That would help elucidate not only what might be driving the fragmentation of tasks but how such fragmentation has the potential to make adjudications of research (mis)conduct and responsibility more complex and difficult. Felipe Romero, in an article that confidently specifies who should perform replication labour (2018), proposes ‘intervening on the social structure of science’ by creating ‘confirmation-research-track positions’ in which researchers would focus entirely on replication work. While Romero discusses how such new positions would demand changes in tenure and promotion guidelines, as well as the role that governments and private funders might play in supporting such positions, he avoids any discussion of the current vicissitudes facing researchers or of the upheavals in and pressures facing the funding of any kind of research position. It is indeed difficult to find writings that bear on questions of replication and reproducibility that manifest sustained interest in the broader political economy of either the sciences or the university (Berman, 2012; cf. Rajan, 2007).

## Research Culture, Transparency and Efficiency

Culture, unlike labour, is explicitly named in multiple writings addressing the replication crisis. Consider, for example, Marcus Munafò and colleagues’ article ‘Research culture and reproducibility’ (2020). In it, they noted that ‘culture is both pervasive and difficult to observe’:

We create our culture, invisible though it may be, and we therefore have it collectively within ourselves to change our culture for the better. Our institutions in particular, as repositories of this culture, can also be crucial in fostering change. (2020, p. 92)

Or consider an article co-authored 5 years earlier by Brian Nosek, another prominent figure keen to reform practices of science in light of his understanding of the replication crisis. That article was titled ‘Promoting an open research culture’ – though the text of the paper did not substantively interrogate the term ‘culture’ (Nosek et al., 2015). Or consider the book by Chris Chambers on the multiple ‘cultural sins’ of the psychological science of psychology (2019, p. 172). Chambers fondly conjures how he has ‘always thought of our professional culture as a [castle] – a sanctuary of endeavour built long ago by our forebears’ but one which the discipline of psychology has ‘allowed ... to fall into a state of disrepair’ (see also Flis, 2019 on this passage). Or consider Scott Lilienfeld, one of the few participants in the replication crisis literature to address the precarity of researchers, who points to this through a language of *culture*: ‘The corporate *culture* of academia places young scholars in a precarious position, as they feel incessant pressure to secure grant funding even if they do not need it [italics added]’ (Lilienfeld, 2017). Or consider the Wellcome Trust, one of the largest grant-giving bodies in science and medicine, which has established a programme and priority area entitled ‘Reimagine Research Culture’, which responds to perceived problems in research ecosystems, including problems of reproducibility (Wellcome Trust & Shift Learning, 2020). Notably, Wellcome frames its project as ‘let’s reimagine how we work together’ (Wellcome Trust, 2020). For me, the question remains: how do we ensure that ‘how we work together’ is as much concerned with the materiality of work as with relations at work? How might such initiatives ensure that concern with the interpersonal (how we work *together*) does not turn things back to being wholly a question of culture rather than one of labour (how we *work* together)?

To understand the growing interest in ‘research culture’ – in and beyond discussions of the replication crisis – we need to turn back to the consolidation of the concept of ‘organizational culture’ in the 1980s. It is the concept of ‘organizational culture’ that provides many of the frameworks through which ‘research culture’ is being thought. Organizational culture literature is deeply indebted to foundational writings by figures such as Hofstede, 1980; Pettigrew, 1979; Schein, 1981. Both Hofstede and Schein wrote as psychologists, and much of this body of work was concerned with the norms, attitudes and values that shape an organization. While the literatures on organizational culture did draw on anthropological and sociological approaches to culture, psychology (and psychological constructs) were central. Here is another exemplification of the circuitry of psychology (Morawski, 2020; Richards, 2002): psychological constructs are woven into conceptualizations of organizational culture, which then shape understandings of research culture, which then are entangled in the approaches scientists and others devise to improve this ‘culture’. In this process, as psychological constructs and formulations move across different spheres and are transformed by them, they help stake out the terms through which a phenomenon, and a problem, is understood. Such circuitry makes it more difficult for other conceptualisations of culture – in which questions of labour might be far more firmly embedded (e.g. Terranova, 2000) – to gain a foothold.

Invocations of research culture also require particular figurations of the scientist. The particular norms the scientist is said to embody display the centrality of the vocational and professionalizing model that was put in place in the 20th century, and which ones finds in the writings of Robert Merton (1957; see also Shapin, 2010). This model casts its shadow over today’s replication crisis. That means that concerns with, and investment in, the vocation and passion of the scientist overshadow interrogations of employment relations and the nature of work. That it is difficult to discern questions of labour – and exploitation, and appropriation – within the replication crisis is in many respects unsurprising. Many have written about the difficulty of acknowledging labour issues in writings about the university. Thomas Discenna has argued that ‘the modern university was founded on the denial of labour through its appeal to culture as a meta-narrative offering legitimacy to the work that goes on within it’ (2018). Christopher Newfield, in tracing the making of the twentieth-century US university, explores the role of professionalized ‘craft labour’. Here, the professional was envisaged as able to define their own work – to control their own conditions of production – and hence, unlike other workers, manifest a kind of freedom (2004). Nick Mitchell has described how tenured academic work frequently does not figure itself as work, but rather ‘continues to occult itself, jealously guarding the sociological vestiges of a privilege derived from its relation to the clerical world’ (2019).

The reticence in the replication crisis literature concerning the potentially pathogenic nature of work can be contrasted to the relative ease of discussions concerning potentially pathogenic elements within the culture of science. Merton, in his well-known essay ‘Priorities in scientific discovery’, stated that ‘the culture of science’, particularly given its enormous emphasis on ‘original discovery’ is, ‘in this measure, pathogenic’ (1957, p. 659). Scientists find themselves in an inherently stressful situation in which original discoveries are demanded and yet elusive: this encourages ‘self-assertive claims, secretiveness lest one be forestalled, reporting only the data that support an hypothesis, false charges of plagiarism, even the occasional theft of ideas and in rare cases, the fabrication of data …’ (1957, p. 659). Here, pathogenesis is thought in relation to a collectivity of individual scientists who make up a scientific culture. As traces of the Mertonian model reappear in discussions of the replication crisis, current incentive systems in science are interpreted as exacerbating these pathogenic tendencies. Potentially pathogenic consequences are understood to arise from these ‘cultural’ features of the research landscape, but there is little interrogation of broader political-economic processes through which such ‘cultural’ features might emerge and intensify. For example, Lilienfeld, in lamenting researchers’ lack of time to ‘think deeply’, links this to today’s ‘today’s supercharged grant environment’ (2017), but does not delve more deeply into how current configurations of an increasingly financialized and digitalized university might be contributing to reorganisations of time and of labour well beyond an incentive system that rewards the getting of multiple grants (see Wajcman, 2015). The 2020 study on research integrity commissioned by UK Research & Innovation does note that employment and workload conditions, particularly the use of precarious contracts, can have a deleterious effect on incentive structures and hence on research integrity (Metcalfe et al., 2020). The report ultimately emphasizes, however, the need for greater support and more ‘positive’ incentives so as to ‘[foster] a culture of continuous improvement’ (Metcalfe et al., 2020). Current conditions of labour are, once again, presented as a trying and intractable background against which the work of cultural transformation might take place.

Similar analytical framings are visible when discussions of the replication crisis turn to the much lauded norms of transparency, openness and efficiency (e.g. Begley & Ioannidis, 2015; Polanin et al., 2020). These terms are abstractions with complex histories that can be understood and have been used in multiple ways (e.g. Strathern, 2000). It is important to note that both virtues not only appear in relation to replication but in university ecologies that that have embraced New Public Management (Lorenz, 2012). While such terms are said to promise multiple benefits for research culture, their practice may well bring many deleterious consequences for research labour. The drive towards efficiency, facilitated by transformations in the mode of production, is accompanied, for John Welsh, by ‘new techniques of appropriation’ that are ‘brought to bear upon the unacknowledged and invisible labourer’ (2020). The commitment towards transparency, underpinned by the reliance on audit and evidence, naturalizes, for Neil Cocks (2017), new forms of authority in an increasingly corporatized university.

The values and virtues of transparency, openness and efficiency require further attention in discussions of the replication crisis. Understanding their potential implications for labour demands getting to grips with how much additional burden – and for whom – is required to make things open and to keep track this openness (Star & Strauss, 1999). Nadine Levin and Sabina Leonelli, in exploring open science in biological research, have emphasized how open science requires different kinds of labour. Only some of these forms of labour are made visible and are valued; many rely on voluntary, invisible and unremunerated labour (2017, p. 284). Many of the infrastructures and practices that support open research and data sharing – whether in the form of coding and software, or the increasing reliance on various platforms to track the registration, stages and impacts of research – are maintained through a mixture of labour that is waged and unwaged, adequately and inadequately remunerated, freely given and exploited. Tiziana Terranova’s research (2000) on how ‘free labour’ produces culture for the digital economy, and Steven Shapin’s essay (1989) on the transparency of the early modern technician to the gaze of his scientific employer beautifully demonstrate how, under certain conditions, both commodities and labourers can become transparent – and hence invisible (Suchman, 1995). Various efforts to increase transparency and efficiency – along with their attendant regulations, modes of surveillance and additional tasks – exacerbate problems for labour (in highly unequal, gendered and racialized ways), and hence intensify the crisis of social reproduction in the university. David Peterson and Aaron Panofsky, for example, in their analysis of metascience as a social movement whose engine is provided by the replication crisis, make clear how ‘efficiency’, as used within the metascience literature, is envisaged as a way of removing the ‘friction’ that slows down research. The aim, here, is once again to create a culture ‘in which self-correction operates efficiently’; they point out that it is assumed that there is ‘little or no cost associated with copying, sharing and hosting data’ (2020). This, we should be clear, is a familiar fantasy: a fantasy of work without workers (McGlazer, 2020).

## Labour in Psychology, the Labour of Psychology

Where might we find labour, then, in discussions of scientific – and specifically psychological – research? There is still much research to do on how changing forms of labour inside and outside the university have shaped, and have also been shaped by, the discipline of psychology.^4^ A special section of *Journal of Social Issues* addresses the ‘neoliberalization of psychological knowledge’: in it Glenn Adams and colleagues argue that the psychological sciences amplify the authority of neoliberal systems (Adams et al., 2019), while Gjorgjioska and Tomicic (2019) outline, using the example of Social Representations Theory, what they regard as a ‘crisis in social psychology under neoliberalism’. While the special section mentions problems of casualization and precariousness, we are still missing fine-grained elaboration of labour practices and how these might contribute to epistemic formations. Indeed, there is a paucity of research that relates issues of labour to psychology as a discipline – a discipline that has certain distinctive features when considered alongside other life and social sciences. One of the aims of this article is, indeed, to encourage additional research that might draw the history and sociology of labour into closer proximity to the history and sociology of psychology.^5^

Psychology is a profoundly heterogeneous, non-unified discipline that is impossible to cohere into one object with one history (Smith, 1988). Today, it stretches from experimental psychology centred on laboratory animals, to exploratory data mining of large data sets, to biophysiological research, to clinical psychological research. It is deeply embedded in many interdisciplines and other disciplines – including the cognitive, behavioural and affective neurosciences, and behavioural economics. It is taught in faculties of education and business as well as health, science and social science faculties. While its methods and epistemological frameworks are also heterogeneous, positivism remains influential in psychology’s present as well as its past consolidation as a discipline. Psychology is notable for its extensive use of psychology students within psychological experimentation (Leentjens & Levenson, 2013) – which raises a number of unresolved questions concerning ethics, coercion and exploitation. Empirical psychological science has a tendency towards small effect sizes and small sample sizes (Maxwell, 2004). There is, currently, growing interest in ‘big team’ psychology: this is envisaged as collaborative, large teams who work across national borders and who aim to combat methodological problems associated with the replication crisis (Moshontz et al., 2018; Forscher et al., 2020)), such as the overreliance on samples that have been drawn from ‘Western, Educated, Industrialized, Rich and Democratic societies’ (Henrich et al., 2010). And we should not forget that psychology, unlike some other disciplines, carries a long and often florid history of named crises that extends back many decades (Morawski, 2019). All these aforementioned features of psychology^6^ merit further analysis, both in relation to discourse concerning the replication crisis, and for their potential contribution to making it difficult to locate labour in discussions concerning knowledge production in this discipline. Given psychology’s many distinctive attributes, it is perhaps surprising that Nicole Nelson and colleagues, in their mixed methods empirical investigation of the discursive dimensions of the replication crisis (2021), found many similarities in the ways in which articles from psychology and articles from biomedicine have discussed the replication crisis. While psychology articles tended to feature more discussions of statistical techniques, and frequently covered the failure to replicate long-standing findings such as ‘priming’ effects, there is, Nelson and colleagues argue, a ‘clear thematic core to reproducibility discussions’. Biomedical scientists and psychologists converge in their assessments both of the sources of replication failure and in their proposed solutions to such failure. And here, once again, Nelson and colleagues find, across biomedical science and psychology, a preoccupation with problems with the *culture* of science.

While it is difficult to locate labour in discussions of psychology, and its histories, directly, several science and technology studies scholars and other social scientists offer fruitful pathways for investigating labour in psychology even if they do not specifically focus on psychology themselves. Such literature probes how epistemological preoccupations and contestations often arise from divisions and reorganizations of scientific labour, and these authors also pull into visibility forms of labour that are left out of many ruminations on psychological experimentation. Park Doing (2004), in examining the work of operatives in science laboratories, has demonstrated how epistemic politics that take the form of arguments over expertise are centrally arguments over labour. Scroggins and Pasquetto (2020) make visible the intense and often invisible labour that makes up data-intensive science and take up Arlene Daniels’ famous essay (1987) on invisible labour as that which ‘disappears from our observations and reckonings’. Importantly, Scroggins and Pasquetto emphasize how little is yet understood about how data-intensive methods might be transforming norms of work. They thereby open trajectories still be explored in assessing how the emergent field of metascience (Peterson & Panofsky, 2020), as well as increasingly data-intensive practices within psychology, might be related to problematics associated with the replication crisis. Scroggins and Pasquetto contrast such invisible work with certain ‘hypervisible work’, which is in part characterized by the enjoyment that is provoked by the observation of such labour; into this category they place replication scandals in social psychology and elsewhere. Here, one might say that certain kinds of labour tied up with the ‘replication crisis’ – namely the labour expended in affectively intense discussions about this named phenomenon – could be seen to blot from view many other forms of labour that are less visible to those preoccupied by this phenomenon, and arguably more central to analyse if one is to understand it. Philip Mirowski (2018, p. 177), in his sharply critical assessment of the Open Science movement, lambasts how citizen science, often lauded by open science advocates, frequently means greater numbers of unremunerated people being drawn into practices of science, thereby ‘primarily serv[ing] as the passive reserve army of labour in the marketplace of ideas’. An emerging body of work directly addresses exploitation (and self-exploitation (Brienza, 2016)) in research. Sukarieh and Tannock (2019), for example, focus on the increasing use of sub-contracted, and exploited research labour, particularly in research that is led by principal investigators in the Global North while being undertaken by research assistants in the Global South. Their argument raises serious questions of epistemology and authorship (what does it mean for publications and data to be authored and authorised by researchers who have not in any way been close to their production, and who at times exclude from authorship the generators of knowledge?). It also addresses head on the political economy of globally produced research. For Sukarieh and Tannock, ‘problems and inequities in the industrial organisation of academic research production’ can only be addressed by a wholesale restructuring of the ‘distribution of wealth, power and control of research agendas and practices among academic researchers’ in the Global South as well as the Global North.

As a starting point for what I hope might become more extensive investigations of what labour in increasingly data-intensive psychological sciences look like today, I offer brief questions to ask in relation to the labour of: (i) researchers; (ii) research participants and (iii) workers in what we might call ‘para-research’ spaces.

### Researchers

How might we produce more finely grained models and accounts of current psychological research and research practice? What forms of labour constitute it, using which kinds of contracts? How might we better understand processes of exploitation, extraction and appropriation (Welsh, 2020)? Have we fully grasped the socio-political as well as epistemic consequences of the ‘new circuitry of academic capitalism’ in which, in many locations, it is fixed-term, postdoctoral labour that does the bulk of the research itself? How do we grapple with those consequences both in terms of the conduct of that work itself, the threats to academic freedom that such conditions pose – and their potential bearing on phenomena associated with the ‘replication crisis’? How exactly might labour relations inflect epistemic cultures as well as ethos? How do political-economic relations within and beyond the university affect disciplinary and interdisciplinary transformations – and vice versa? How exactly does the costing of research labour through time-pressured grants shape how research is done? Which labour remains unremunerated and how does this affect epistemic cultures? How do new divisions of research labour, or the emergence of new practices of variously skilled work, affect how data are collected, compared, gathered, or how experiments are done and recorded? How do forms of hierarchy in academic labour intertwine precarity with other forms of dependency (e.g. Peacock, 2016), and what consequences does this have epistemically as well as socially?

### Research Participants

How might psychology better conceptualise research participation as labour?Waldby and Cooper (2014), pp. 7–8 have emphasised that one of the central historical missions of bioethics – the discipline that has maintained epistemological and procedural oversight over research participation – has been to protect the research participant from market forces. This has made it far harder to protect the research participant through formal instruments of twentieth- (and now twenty-first) century labour law. Waldby and Cooper push against this sequestration of research participation from labour, through articulating a labour theory of value for clinical labour, which they regard as a crucial element of bioproduction (see also Waldby & Cooper, 2008). Their interest in thinking research participation as a mode of work is shared with those such as Jill Fisher (Fisher et al., 2020) and Roberto Abadie (2019), for whom research ‘guineapigs’ experience various kinds of exploitation similar to others who are captured by the precarious gig economy. How might such research open up how such labour processes might bear on data reliability and validity – and hence on the phenomena under investigation by those preoccupied by problems of replication?

It is not, moreover, simply in relation to clinical trials in which the bodies, as well as the minds, of research participants, are centrally at stake. Labour exploitation might be found in multiple modalities of psychological research being conducted today. In addition to the ongoing use of undergraduate student labour in psychological experimentation, does it matter that recent years have witnessed an enormous rise in psychological studies that use Amazon’s Mechanical Turk (MTurk) (Buhrmester et al., 2018; Chmielewski & Kucker, 2020)? While some academic articles, often from disciplines outside of psychology, address labour exploitation and MTurk (Pittman & Sheehan, 2016), few within psychology do. And yet it is certainly possible that the labour conditions of MTurkers might bear on questions of data reliability and validity that are raised by those concerned about a replication crisis. Notably, the only research article I have located that discusses the use of MTurkers in relation to psychology and data quality focuses on how best to weed out bots (programs that complete human intelligence tasks automatically) and farmers (individuals who deploy server ‘farms’ to side-step location restrictions specified within MTurk) (Chmielewski & Kucker, 2020). The only mention of payment in that article is in the context of paying ‘genuine’ MTurkers promptly so as to aid the process of weeding out illegitimate bots or farmers. Here, once again, the question of labour – both the labour of the poorly paid MTurkers, and the political-economic processes that might help account for the proliferation of bots and farmers – is rendered invisible.^7^

### Para-Research Spaces

How far can the vision of the Mertonian collectivity of scientists, who are put under pressure by the need for original discoveries, take us in understanding the production of the (increasingly data-intensive) psychological sciences today? How might we better enumerate, make visible and include in our analyses of the replication crisis the multiple forms of labour that make up not only experimentation but research audits, research dissemination and practices of research governance? How do we grapple with the frequently invisible, and highly racialized labour relations that underpin the production of databases, the tagging of content, the scanning of materials into Google and other for-profit ‘knowledge providers’ – all of which are a core component of research culture today (see Cohen, 2019)? How do the growing numbers of for-profit providers (Holmwood & Servós, 2019) and the intensification of market activities affect what kinds of psychological research are conducted, how, and by whom? How might a continuing push towards ‘big team science’ in psychology facilitate the emergence of new kinds of labour requirements relating to the sourcing, management, and analysis of data? What kinds of relations between those in the Global South and Global North are enabled by ‘big team science’?

The replication crisis in psychology is repeatedly framed as a crisis involving actors who are, for the most part, simply named as ‘psychologists’. But once one has enumerated the varied forms of labour that make up today’s assemblage of psychological research, use of the generic term ‘psychologist’ to denote the main actor in the field starts to seem obfuscatory, rather than acting as a helpful shorthand. Which actors are most visible in debates concerning the replication crisis? Who fails to reach visibility? Which kinds of psychologists in which kinds of employment contracts come to exemplify the figure of the ‘psychologist’? From which epistemic standpoints, gathered in which kinds of labour relations, is knowledge about the replication crisis being produced? In thinking through the stakes of these questions, we would do well to heed Eli Thorkelson, who argues, in their essay on the American university sector, with its extensive reliance on casualised labour, that ‘there is no universal subject in the American university, no group structurally able to aspire to epistemological totality’ (2014, p. 2). In the replication crisis literature in psychology, there does tend to be an imagined ‘universal subject’ able to wield epistemological totality: the psychologist. This figure takes on a fantasy-mask: she frequently acts as a kind of Mertonian figure, one in which one finds a particular entwinement of ethos and epistemology. But in the wings, off stage, are multiple other figures who complicate this fantasy. Those who are keen to ameliorate research cultures or to resolve the replication crisis frequently use a language of the ‘we’. Munafò and colleagues (2020, p. 92), for example, when discussing research culture, write about how ‘we’ can change it. But who exactly is able to be held within the apparent generous embrace of this first person plural? In their article they invoke the ‘community’ – the ‘reviewers, editors, panel members and so on’. But there are relatively few workers, of the many that make up the domain of scientific research, that do indeed have the capacity to ‘change’ this culture, rather than simply be subjected, in the workplace, to particular cultural-economic configurations which they have little possibility of shifting. Which kinds of change, moreover, are possible, if one envisages a community of researchers, largely divorced from material conditions (see Carpenter et al., 2021)? The history of labour tells us while certain changes to working cultures can be achieved through transformations in conditions and governance that are taken on by certain elements of ‘the community’, or encouraged by the guiding hand of research funders such as the Wellcome Trust, most have required unionization and labour disputes. The term ‘psychology’ figures in particular ways in the replication crisis literature, ways that betray a reliance on the language and fantasy of professionalisation.

The task in front of us today is to ask to what extent such a scene comprising a collectivity of individual, professionalized psychologists (aided by the wondrous tools of data science) accurately describes how psychology is made. This scene might be more accurately described as a scene made up of multiple forms of labour, and characterized by under-analysed forms of exploitation and appropriation that point to an intensifying crisis of social reproduction.

## Conclusion

The post-2008 moment, in psychology specifically and in the university more broadly, is one in which many have said there are failures of replication and of reproduction. Are there crises, now, in psychology and in academic labour, or is it, rather, that there are currently extensive discourses of crisis? The title of this article, ‘Replication and reproduction: crises in psychology and academic labour, is ambiguous. Like several who have written about the history of psychology or the history of the university (Boggs & Mitchell, 2018; Morawski, 2019; Moten & Harney, 1999), I resist the inference which is often sparked by today’s crisis language: namely, that if today there is crisis, yesterday there was not. Both the history of psychology and the history of the university contain many instances of phenomena that can be understood as manifestations of crisis. In relation to the university specifically, Moten and Harney’s injunction to ‘realize that academic labour is perpetually in crisis and flux’ is an important bulwark against fantasies of equable labour relations in the twentieth-century university (see also Boggs & Mitchell, 2018; Moten & Harney, 1999, p. 24). Much of the replication crisis debate proceeds as though economic forces and constraints press on the university from the outside. Addressing today’s crisis of social reproduction in the university, alongside other sites for the production of knowledge, needs to ensure that the university is understood to be a dynamic and powerful contributor to, rather than defensive bulwark against, capitalist transformations that come from ‘outside’ (Bacevic, 2018; Baldwin, 2020).

How failures of replication and reproduction are identified, named and analysed matters. They matter for how psychology narrates its history and its present, and they matter for how such a narration contributes to making psychology’s present and future. Extensive discussions of the apparent crisis of replication have found little room for deliberation over the current shape of crisis besetting academic reproduction. The multiple workers – both inside and beyond research institutions – who run experiments, offer their mental and bodily labour as participants in those experiments, make data digital (Cohen, 2019), and much more, are largely not considered as actors in the governing stories through which psychology tells its history and present. Psychology’s epistemological frameworks have tended not to be grounded in the position or experience of the worker: American industrial-organizational psychology, Michael Zickar (2004) has argued, has shown a general indifference to labour unions and has had a tendency to identify with management. The hypervisibility of the work of making the replication crisis a phenomenon – a crisis! – is accompanied by the invisibility, in discussions of that phenomenon, of much of the labour that makes up the psychological sciences. This creates an imaginary in which much of the work of the psychological sciences is imagined as taking place without workers. While I started my deliberations here by considering the potential salience of the economic crisis of 2007–8 in relation to the emergence of the replication crisis, I consider the events of 2007–8 as an exemplary case through which to consider how labour conditions are imbricated in knowledge production in psychology more broadly. The economic crisis might have brought a crisis in social reproduction in the university into more obvious relief, but it certainly did not single-handedly bring about the practices, working conditions – and culture – of the psychological sciences today.

That questions of ‘culture’ have been central to discussions of the replication crisis but not questions of labour takes us back to enduring preoccupations within the academy from the 20th century. Heather Steffen’s research documents different ideologies of labour that have characterised the twentieth-century US university. She identifies four common ideologies found in critical and narrative writing about the university: the professional, unionist, vocational and entrepreneurial. Different ideologies provide different ways for researchers to imagine ‘their relationships to the institutions, social structures and political economies that constrain and enable research, education and scholarly public service’ (Steffen, 2020). The replication crisis is largely conducted using models and registers of the professional, the vocational and the entrepreneurial. What Steffen calls the ‘unionist’ (a model in which multiple forms of activity in the university are considered as labour) rarely surfaces.

But if the post-2008 moment is to be described as marked by failures of replication and reproduction, it is also, in a certain sense, mired too in particular kinds of *re-production*. For re-production, as the sociologist Sarah Franklin (2015) has argued, points, through a Marxist-feminist lens, to the ‘conditions of consciousness’ that keep in place the reproduction of existing institutional and social modalities. And such modalities frame and constrain what becomes possible and impossible in terms of the working conditions of the academy. Replication crisis talk, in its avoidance of discussions of labour, removes from view that which might significantly influence how the production and re-production of knowledge (including replication) take place.

Much of what is being re-produced, as psychology narrates its present, invokes its history, and re-makes itself, makes it difficult to perceive, let alone to understand, the current contours of today’s crisis of social reproduction in the university. Many within and beyond psychology have been preoccupied with a failure to replicate, even as they find it difficult to acknowledge the labour and infrastructural conditions through which experiments are reproduced. What becomes visible, and what remains invisible, when there is said to be a crisis of replication in and beyond psychology? The visible helps frame what is perceived as a problem and hence shapes the forms of intervention that are created to address it. I believe we need different analyses both of the problems besetting psychology, today, and different imaginaries for how we might address them. This article hopes to contributes to such work. It does so by thinking hard not only about what fails to be reproduced, but also what continues to be reproduced, when some become preoccupied with a failure to replicate.

## Notes

1 *Oxford English Dictionary*, s.v. Replication; reproduction.

2 The phrase reproducibility crisis is also used, and there is ongoing debate over whether the terms replicability and reproducibility should be distinguished from one another in relation to discussions around scientific practice (see Nelson et al., 2021).

3 The highly financialized UK and US university sector comprises the primary focus of my thinking in this article, though many of the arguments are, I believe, relevant beyond those countries.

4 Psychology is not the only discipline in which it is difficult to find discussions of labour conditions. Zheng (2018), for example, has argued in relation to the discipline of philosophy, that the precarious nature ‘of academic employment is the most pervasive and urgent problem confronting the discipline – and the rest of the academy – today’, and wonders why there has been so little attention paid to it.

5 Note in this regard the 2022 Gordon Cain conference, ‘Let’s Get to Work: Bringing Labour History and the History of Science Together’ https://www.sciencehistory.org/event/gordon-cain-conference-2022.

6 My thanks to one of the anonymous reviewers for suggesting several of these.

7 A pre-print whose first author is affiliated to a for-profit participant-sourcing platform for online research and surveys (the platform offers a ‘MTurk Toolkit service’ that promises to ‘totally transform how you use Amazon’s micro-tasking platform’ (CloudResearch, 2015)) concludes that there is not a significant problem with labour conditions using MTurk (Moss et al., 2020).
